# Deubiquitylating enzyme USP9x regulates hippo pathway activity by controlling angiomotin protein turnover

**DOI:** 10.1038/celldisc.2016.1

**Published:** 2016-03-29

**Authors:** Hung Thanh Nguyen, Diana Andrejeva, Rajat Gupta, Chunaram Choudhary, Xin Hong, Pieter J A Eichhorn, Anand C Loya, Stephen M Cohen

**Affiliations:** 1 Department of Cellular and Molecular Medicine, University of Copenhagen, Copenhagen, Denmark; 2 The Novo Nordisk Foundation Center for Protein Research, University of Copenhagen, Copenhagen, Denmark; 3 Cancer Center, Massachusetts General Hospital, Harvard Medical School, Charlestown, MA, USA; 4 Cancer Science Institute of Singapore and Department of Pharmacology, Yong Loo Lin School of Medicine, National University of Singapore, Singapore, Singapore; 5 Department of Pathology, Rigshospitalet, Copenhagen, Denmark

**Keywords:** Hippo pathway, LATS, protein degradation, YAP, ubiquitin

## Abstract

The Hippo pathway has been identified as a key barrier for tumorigenesis, acting through downregulation of YAP/TAZ activity. Elevated YAP/TAZ activity has been documented in many human cancers. Ubiquitylation has been shown to play a key role in regulating YAP/TAZ activity through downregulation of a number of Hippo pathway components. Several ubiquitin ligase complexes have been implicated in this process, however, little is known about the deubiquitylating enzymes that counteract these activities to regulate YAP/TAZ. Here we identify the deubiquitylating enzyme USP9x as a regulator of YAP/TAZ activity. We demonstrate that USPx regulates ubiquitin-mediated turnover of the YAP inhibitor, Angiomotin. USP9x acts to deubiquitylate Angiomotin at lysine 496, resulting in stabilization of Angiomotin and lower YAP/TAZ activity. USP9x mRNA levels were reduced in several cancers. Clinically, USP9x mRNA levels were reduced in several cancers with low USPx expression correlating with poor prognosis in renal clear cell carcinoma. Our data indicate that USP9x may be a useful biomarker for renal clear cell carcinoma.

## Introduction

Regulation of the transcriptional co-activators Yes-Associated Protein 1 (YAP1) and transcriptional coactivator with PDZ-binding motif (TAZ) is a key output of the Hippo signaling pathway in control of cell proliferation, tissue repair and in tumor progression (reviewed in reference 1). YAP and TAZ bind to transcription factors including transcriptional enhancer factor TEF-1 (TEAD), β-catenin and RUNX family members, to regulate genes required for cell proliferation and survival (reviewed in reference 2). Increased YAP/TAZ expression through gene amplification or epigenetic regulation, as well as increased YAP/TAZ activity by mutations in upstream Hippo pathway components have been identified in human cancers (reference 3, reviewed in references 4, 5). Increased YAP activity has recently been reported to replace the requirement for oncogenic K-Ras in models of pancreatic and colon cancer, and in the transformation of primary human cells to create cancer cells [6–8]. YAP expression also contributes to the acquisition of resistance to RAF and MEK-targeted cancer therapies [9]. By limiting YAP/TAZ activity, the Hippo pathway serves as a barrier to cellular transformation. This negative regulation by Hippo can be abrogated through concomitant expression of oncogenic Ras [8], the viral small T oncoprotein [10] or the Kaposi sarcoma-associated herpes virus [11]. As a consequence, the Hippo pathway is currently considered a therapeutic target in cancer and several clinical trials have been initiated to systematically analyze the effects of YAP/TAZ inhibition on tumor progression ([5, 12], ClinicalTrials.gov Identifier: NCT02347163).

Several lines of evidence suggest that regulation of Hippo-pathway protein turnover may play an important role in cancer. The core Hippo pathway is a kinase cassette comprised of the Mammalian sterile-20-like (MST1/2) and large tumor suppressor kinase 1/2 (LATS1/2) kinases. LATS kinases are activated by MST leading to LATS-dependent phosphorylation of YAP and TAZ. YAP and TAZ are subsequently targeted for degradation by the βTrCP/SCF ubiquitin ligase system [13, 14]. We have recently reported that YAP protein turnover is regulated by the Ras pathway, through regulation of the SOCS5/6 proteins, which serve as substrate recognition factors recruiting YAP to an elongin B/C-Cullin5 ubiquitin ligase complex [8]. While destruction of YAP and TAZ is central to the tumor suppressor activity of the Hippo pathway, evidence is emerging that ubiquitin-mediated protein turnover acts at multiple nodes of the Hippo pathway. The RING ligase PRAJA2 induces proteasome-mediated degradation of MOB1, a regulator of LATS kinases, and has been demonstrated to contribute to the pathogenesis of glioblastoma [15]. The E3 ubiquitin ligase ITCH has been shown to regulate the abundance of LATS1 kinase. As a consequence of increased destruction of LATS1, ITCH overexpression is sufficient to promote tumorigenesis [16].

The importance of ubiquitylation in regulation of Hippo-pathway activity prompted us to explore the potential roles of deubiquitylating enzymes (DUBs) as regulators of Hippo pathway activity. The activity of ubiquitin ligases in promoting protein turnover can be counteracted by DUBs, which catalytically remove ubiquitin moieties from proteins. In a few instances, DUBs have been found differentially expressed or genetically altered in human cancers, suggesting their potential roles as oncogenes and tumor suppressors [17–20]. Here, using a cell-based RNAi screen for YAP/TAZ activity, we have identified the DUB USP9x as a negative regulator of YAP/TAZ activity. We report that USP9x regulates YAP/TAZ activity indirectly by regulating the protein stability of the YAP/TAZ inhibitor, Angiomotin (AMOT). AMOT inhibits YAP/TAZ activity through direct physical association with YAP thereby limiting YAP nuclear localization [21, 22], and AMOT has been shown to be targeted for degradation by a Nedd4 ubiquitin ligase complex [23]. These findings provide a molecular framework for the previous observation that USP9x is downregulated in pancreatic ductal adenocarcinoma (PDA) [20] and for the finding, presented here, that low levels of USP9x expression correlate with poor prognosis in renal clear cell carcinoma (ccRCC).

## Results

### Identification of DUBs regulating YAP/TAZ activity

**Figure 1:**
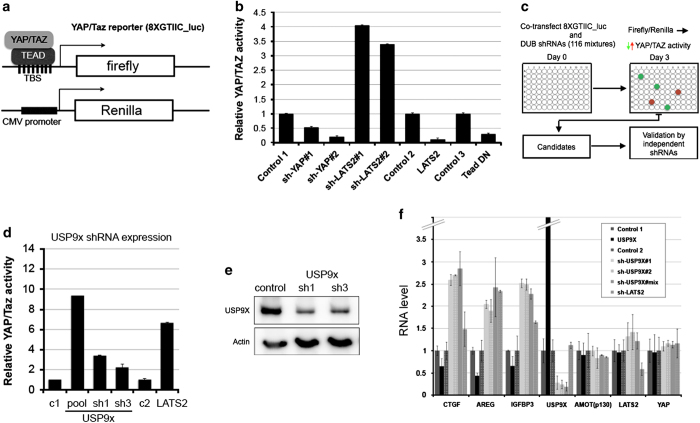
Identification of USP9x as a regulator of YAP activity. (**a**) The 8×GTIIC_luc YAP/TAZ reporter contains eight TEAD-binding sites, to control expression of firefly luciferase. CMV-Renilla luciferase provides a control to normalize for transfection efficiency. YAP/TAZ activity was determined by the ratio of firefly/Renilla luciferase after co-transfection of the two plasmids. (**b**) Luciferase reporter assays showing the effects of changes in Hippo pathway activity. HEK293T cells were transfected to express the luciferase reporters together with shRNA vectors to deplete YAP, LATS2 or with control shRNAs. Cells were also transfected to overexpress LATS2 or a dominant negative form of TEAD vs appropriate empty vectors as controls. Data represent the mean of three independent transfection experiments±s.d. (**c**) Summary of the RNAi screen workflow. (**d**) Luciferase reporter assays showing the effects of changes in USP9x activity. HEK293T cells were transfected to express the firefly and luciferase reporters together with an shRNA pool and two individual shRNAs targeting USP9x. Data represent the mean of three independent replicates±s.d. (**e**) Immunoblot showing the efficacy of shRNA-mediated depletion of USP9x protein. Upper panel probed with anti-USP9x. Lower panel probed with anti-Actin to control for loading. (**f**) Quantitative PCR was used to measure YAP target transcript levels in HEK293T cells transfected to express the indicated shRNAs. USP9x was depletion using the shRNA pool and two individual shRNAs. shRNA-mediated depletion of LATS2 was used as a control for the effect of increasing YAP/TAZ activity. RNA levels for UAP9x and LATS2 are shown to monitor shRNA efficiency. To test the effect of USP9x overexpression, HEK293T cells were transfected to express V5-tagged USP9x or with an empty vector control. RNA was harvested 36 h after transfection. GAPDH was used for normalization. YAP target levels increased, while YAP mRNA was unchanged. Data represent the average of three independent experiments±s.d.

To identify DUBs that affect YAP/TAZ activity, we used a luciferase reporter containing eight copies of the TEAD DNA-binding sequence (8×GTIIC, [24]). HEK293T cells were transfected to express the 8×GTIIC-firefly luciferase reporter and a Renilla luciferase control vector for normalization (Figure 1a). As controls, we verified that short hairpin RNA (shRNA)-mediated depletion of YAP reduced 8×GTIIC firefly luciferase reporter activity; that overexpression of LATS2 or a dominant negative form of TEAD [12] reduced reporter activity and that shRNA-mediated depletion of LATS2 increased reporter activity (Figure 1b). Having confirmed that the reporter was sensitive to manipulation of Hippo pathway activity, we screened 116 shRNA pools designed to target 94 DUBs [19, 25] (Figure 1c). Several shRNA pools affected YAP/TAZ activity by more than threefold in at least three independent experiments (Supplementary Table S1). The shRNA pool targeting USP9x reproducibly increased TEAD reporter activity (Figure 1d). This finding was confirmed using two independent shRNAs targeting USP9x (Figure 1d and e). As a second test of the effects of USP9x depletion, we examined a panel of known YAP/TAZ target genes [26, 27]. Expression of AREG, CTGF and IGFBP3 increased in cells treated with the shRNA pool or with the individual shRNAs (Figure 1f). Reciprocally, expression of these transcripts decreased in cells overexpressing USP9x (Figure 1f). Activation of YAP/TAZ is required for anchorage independent growth of primary human cells [10]. Depletion of USP9x increased soft agar colony formation in HEK293T cells (Supplementary Figure S1). These three lines of evidence suggest that USP9x normally functions to limit YAP/TAZ activity.

### USP9x targets AMOT and AMOT-like proteins

**Figure 2:**
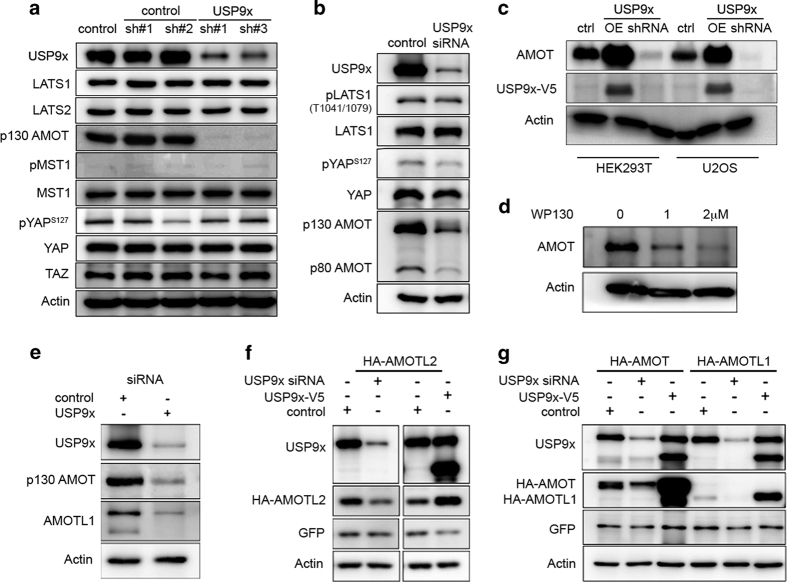
USP9x regulates AMOT levels. (**a**) Immunoblots of HEK293T cells transfected with shRNAs to deplete USP9x or with control shRNAs or with an empty vector control. Blots were probed with the indicated antibodies. Anti-USP9x was directed to the C terminus of the protein and recognizes the full length protein. Anti-Actin was used to control for loading. Note the low level of p130AMOT in USP9x-depleted cells. (**b**) Immunoblots of HEK293T cells transfected with a pool of siRNAs to deplete USP9x or with control siRNAs and probed with the indicated antibodies. (**c**) Immunoblots of HEK293T and U2OS cells transfected to express HA-tagged AMOT p130 together with V5-tagged USp9x (OE) or with a pool of shRNAs to deplete USP9x. Blots were probed with anti-HA to detect AMOT, anti-V5 to detect USP9x and anti-Actin as a loading control. (**d**) Immunoblots of HEK293T cells treated with the DUB inhibitor WP130 at the indicated concentrations. Blots were probed with endogenous AMOT and with anti-Actin as a loading control. (**e**) Immunoblots of HEK293T cells transfected with a pool of siRNAs to deplete USP9x or with control siRNAs and probed with antibodies to p130AMOT and USP9x (C terminus). (**f**, **g**) Immunoblots of HEK293T cells transfected to express HA-tagged p130-AMOT, AMOT-L1 or AMOT-L2 together with a pool of shRNAs to deplete USP9x or with V5-tagged USp9x (or empty vector control). Blots were probed with anti-HA to detect the AMOT family proteins, with anti-USP9x and with anti-Actin as a loading control. Antibody to the N terminus of USP9x detects two forms of the protein. The slower migrating form is also detected by antibody specific to the C terminus of the protein. The faster migrating form is a cleavage product, which has DUB activity (Supplementary Figure S2b). Cells were co-transfected to express GFP, and blots probed with anti-GFP as a control for transfection efficiency. Depletion of USP9x did not reduce AMOT, AMOTL1 or AMOTL2 mRNA levels (Supplementary Figure S3).

To determine how USP9x affects YAP/TAZ activity, we examined the expression levels of elements in the Hippo pathway (Figure 2a). HEK293T cells were transfected to express shRNAs targeting USP9x and with a scrambled control shRNA. An increase in YAP/TAZ activity could result from an increase in the amount of Yap or TAZ proteins, or from a change in the activity of the upstream Hippo pathway kinases MST and LATS1/2. We did not observe a significant change in the amount of YAP or TAZ protein (Figure 2a and b). Increased YAP activity could result from reduced LATS-mediated phosphorylation. However, there was no reduction in phosphorylation of YAP on S127, a readout of LATS activity (Figure 2a and b). Consistent with this, there was no change in the level of the LATS1 or LATS2 kinases, or in the phosphorylation status of LATS1 (Figure 2a and b). Nor was there any discernable change in the level or phosphorylation status of the upstream LATS kinase, MST1 (Figure 2a). Together, these findings suggest that the change in YAP activity resulting from depletion of USP9x is not mediated via regulation of the Hippo pathway.

This prompted us to explore other mechanisms of regulating YAP/TAZ activity, in search of USP9x targets. AMOT has been identified as a regulator of YAP/TAZ activity [21, 22]. The p130 form of AMOT binds to YAP/TAZ, and when overexpressed p130-AMOT can reduce YAP/TAZ activity. Conversely, depletion of AMOT leads to elevated YAP/TAZ activity. We found that endogenous AMOT protein levels were strongly reduced in cells depleted of UPSP9x (Figure 2a and b). Both the p130 and p80 forms of AMOT were affected (although p80 is not thought to regulate YAP activity). To test the effects of USP9x overexpression, HEK293T cells and U2OS cells were transfected to express USP9x-V5-tagged and hemagglutinin (HA)-tagged p130-AMOT. Overexpressed USP9x increased the level of p130-AMOT (Figure 2c). We also confirmed that shRNA-mediated depletion of USP9x reduced HA-tagged p130-AMOT in U2OS cells (Figure 2c). As an independent means to test the effect of reduced USP9x activity, we treated cells with the small molecule DUB inhibitor, WP130 [28]. WP130 treatment led to reduced AMOT protein levels (Figure 2d). Together, these findings provide evidence that USP9x can regulate AMOT protein levels.

AMOT is a member of a family of related proteins including AMOTL1 and AMOTL2 [21, 22, 29]. To ask whether USP9x has a similar effect on the levels of the other family members, we treated HEK293T cells with siRNAs to deplete USP9x and monitored the level of endogenous AMOT-like proteins by immunoblotting. Endogenous AMOT and AMOTL1 were reduced in cells depleted of USP9x (Figure 2e). Endogenous AMOTL2 was not detectable with the available antibodies. To examine AMOTL2, HEK293T cells were transfected to express HA-tagged AMOTL2. HA-AMOTL2 levels decreased in cells depleted of USP9x (Figure 2f). Reciprocally, HA-AMOTL2 levels increased in cells overexpressing V5-tagged USP9x (Figure 2f). Comparable results were obtained with HA-AMOTL1 (Figure 2g). Thus all AMOT-like family members appear to be similarly regulated by USP9x.

**Figure 3:**
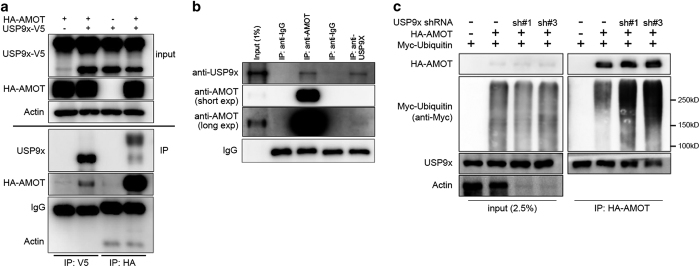
USP9x regulates AMOT ubiquitylation. (**a**) Immunoblots of HEK293T cells transfected to express HA-AMOT and V5-tagged USP9x. Cell lysates were immunoprecipitated using anti-HA to pull down HA-p130-AMOT or with anti-V5 to pull down V5 AMOT. Blots were probed with anti-V5 to detect USP9x-V5 (input) and with anti USP9x (IP). Note that the V5 antibody pulls down predominantly the shorter form of USP9x. Both long and short forms of USP9x were recovered by IP with HA-AMOT. (**b**) Immunoblots of HEK293T cell lysates immunoprecipitated using anti-AMOT or anti-USP9x or with non-immune IgG as a control. Blots were probed with anti-AMOT and with anti-USP9x. The recovery of endogenous AMOT by IP was very efficient. However, recovery of UPS9x by IP was very weak, which limits the sensitivity of detection of AMOT by USP9x IP. (**c**) Immunoblots of HEK293T cells transfected to express HA-AMOT with Myc-tagged Ubiquitin, and shRNA vectors to deplete USP9x or control shRNAs. Cells were treated with MG132 to block proteasome activity. Lysates were immunoprecipitated with anti-HA to recover AMOT and blots were probed to detect Ubiquitin (anti-Myc), AMOT (anti-HA) or with anti-Actin to control for loading. Anti-USP9x was used to monitor the efficiency of shRNA-mediated depletion in the total cell lysate. Depletion of USP9x was efficient. Note that the total level of ubiquitylated protein in the cell was unchanged in the total cell lysates after USP9x depletion. IP, immunoprecipitation.

To ask whether USP9x could potentially act directly on AMOT proteins, we performed co-immunoprecipitation (IP) assays. IP of HA-AMOT recovered USP9x from HEK293T cells co-transfected to express both proteins (Figure 3a). Reciprocally, IP of V5-USP9x recovered HA-AMOT (Figure 3a). Endogenous USP9x protein was recovered by co-IP with endogenous AMOT protein using anti-AMOT (Figure 3b). Recovery of endogenous USP9x protein by IP was relatively inefficient, presumably due to poor performance of the antibody in IP experiments. Nonetheless, on longer exposure it was possible to detect co-IP of endogenous AMOT with USP9x (Figure 3b). HA-tagged forms of AMOT, AMOT-L1 and AMOT-L2 were recovered by IP with endogenous USP9x (Supplementary Figure S2a). These experiments provide evidence that USP9x can physically interact with all the AMOT family members.

If USP9x act directly on AMOT, we would expect to see elevated levels of ubiquitylated AMOT in cells depleted of USP9x. HEK293T cells were transfected to express HA-tagged p130-AMOT and Myc-tagged Ubiquitin with or without shRNAs targeting USP9x. Depletion of USP9x increased the incorporation of ubiquitin into AMOT (Figure 3c). These findings are consistent with a model in which USP9x-mediated deubiquitylation increases AMOT stability. Consistent with this, we observed that the effects of SUP9x on AMOT stability were blocked in cells treated with the proteasome inhibitor MG132 (Supplementary Figure S3).

### Lysine 496 is a key target of USP9x in regulating AMOT turnover

**Figure 4:**
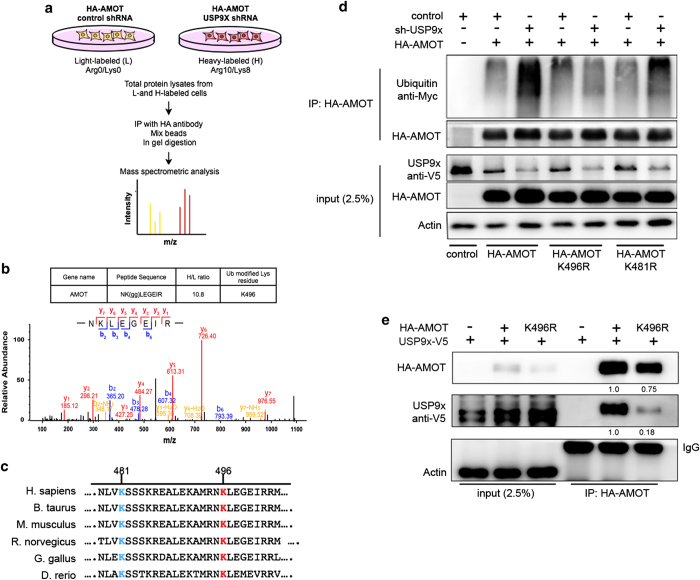
K496 is required for USP9x activity on AMOT. (**a**) Design of the SILAC-based mass spectrometry experiment to detect ubiquitylation sites in AMOT. (**b**) Annotated tandem mass spectrum of ubiquitylated (di-Gly modified) peptide corresponding to AMOT K496. (**c**) Sequence alignment of the region of AMOT containing residues K481 and K496 from the indicated species. (**d**) Immunoblots of HEK293T cells transfected to express HA-tagged AMOT p130 with Myc-tagged Ubiquitin and shRNA vectors to deplete USP9x or control shRNAs. Three forms of HA-AMOT were tested: native, K496R and K481R mutant. Cells were treated with MG132. Lysates were immunoprecipitated with anti-HA to recover AMOT and blots were probed to detect Ubiquitin (anti-Myc), AMOT (anti-HA) or with anti-Actin to control for loading. Anti-USP9x was used to show the efficiency of shRNA-mediated depletion in the total cell lysate. (**e**) Immunoblots of HEK293T cells transfected to express HA-tagged native AMOT or K496R mutant AMOT together with V5-tagged USP9x. Cells were treated with MG132 before harvesting cell lysates for immunoprecipitation with anti-HA. Blots were probed with anti-HA to detect AMOT and with anti-V5 to detect USP9x. The HA-AMOT and V5-USPx bands were analyzed using Image J (www.NIH.GOV) and the values are shown below, with the AMOT-K496R mutant lane normalized to the adjacent native AMOT lane. K496R AMOT expression was somewhat lower than the native protein, as shown in the total lysate panel (at left).

To investigate how USP9x interacts with AMOT, we performed SILAC-based mass spectrometric analysis on AMOT protein isolated from cells depleted of USP9x by shRNA treatment. The design of the SILAC experiment is shown in Figure 4a. Mass spectrometric analysis of AMOT identified a ubiquitylated peptide corresponding to lysine residue K496 in p130-AMOT (Figure 4b). AMOT has previously been reported to be ubiquitylated on K481 by Atrophin-1 Interacting Protein 4 [30]. Both K496 and the adjacent K481 reside in a highly conserved sequence string found in all vertebrate AMOT proteins (Figure 4c). Ubiquitylation of K496 has not been described previously. Supplementary Figure S4 shows alignment of the corresponding region with AMOTL1 and AMOTL2.

To explore the relevance of K496, we asked whether mutating K496 to Arginine would affect the ability of USP9x to deubiquitylate AMOT. HEK293T cells were transfected to express native or K496R mutant HA-p130-AMOT and Myc-tagged Ubiquitin constructs together with a vector expressing control shRNA or USP9x-specific shRNAs. AMOT was immunoprecipitated using anti-HA and the blot was probed to visualize Myc-tagged Ubiquitin (Figure 4d). Depletion of USP9x increased the amount of Ubiquitin recovered on native AMOT, but had little or no effect on the amount of ubiquitylated K496R AMOT protein (Figure 4d). K481R AMOT was sensitive to USP9x depletion, though less than native AMOT (Figure 4d). Proteome-wide ubiquitylation studies have described a number of candidate ubiquitylation sites for p130AMOT including K94, K156, K206, K215, K230, K255, K355, K520, K535, K585 and K619 [31, 32]. Mutation of these Lysine residues to Arginine had little or no effect on the sensitivity of the mutant forms of AMOT to changes in USP9x expression (Supplementary Figure S5a).

These findings suggest that K496 is a functionally significant target of USP9x action on AMOT. To ask if binding of USP9x to AMOT depends on K496, HEK293T cells were transfected to express HA-tagged AMOT or AMOT-K496R together with V5-tagged USP9x, and cell lysates were immunoprecipitated with anti-HA to recover AMOT protein. Recovery of USP9x was considerably reduced by replacement of the Lysine residue at K496 with Arginine (Figure 4e; ratio=0.24 after correcting for the reduced amount of K496R recovered in the IP). This suggests that the absence of lysine at position 496 as a potential substrate reduced interaction with USP9x. Consistent with this, AMOT-K496R was considerably less sensitive to proteasome-mediated degradation than the native protein (Supplementary Figure S5b). Together, these findings suggest that K496 is an important target site through which USP9x acts on AMOT to regulate YAP/TAZ activity.

### Low USP9x correlates with poor outcome in ccRCCs

Recent literature on the role of USP9x in cancer is equivocal. One report has presented evidence that USP9x can promote tumor cell survival through stabilization of the pro-survival BCL2 family member MCL1 in human follicular lymphomas and diffuse large B-cell lymphoma and multiple myleomas [33]. A second report identified USP9x as a tumor suppressor in a K-RAS mouse model of PDA [20]. In this report, PDA patients with low USP9x levels showed poor survival and were more likely to have metastatic disease compared with other patients. Deletions or mutations in USP9x have been identified in 4% of sequenced pancreatic adenocarcinomas in the Cancer Genome atlas (TCGA) PAAD study (www.cbioportal.org), but none were found in another study [34].

**Figure 5:**
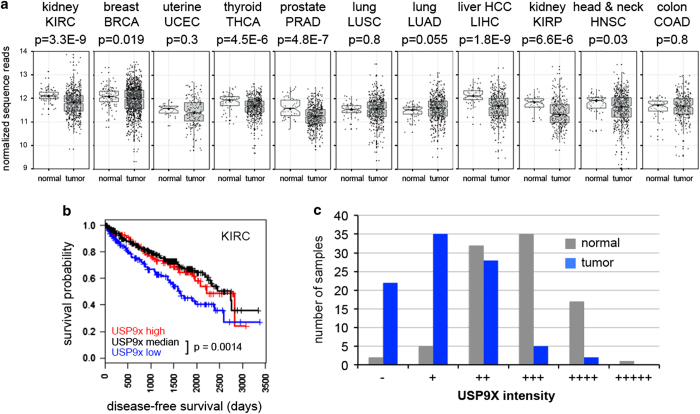
USP9x levels correlate with poor outcome in ccRCC. (**a**) Analysis of USP9x mRNA level comparing normal control tissue and tumor samples from the indicated TCGA data sets. Gene expression values were normalized to a fixed upper quartile value of 1 000 using RNA-Seq by Expectation maximization and represented as scatter plots. Statistical significance was determined using Mann–Whitney tests. (**b**) Plot of disease-free survival for ccRCC patients as a function of USP9x transcript level. Blue denotes patients with bottom quartile mRNA expression; red denotes patients with top quartile expression and black denotes the middle 50% of patients. A new tumor diagnostic indicator or death was scored as events. Cox proportional hazards regression model was used to calculate *P*-values. (**c**) Plot of the histopathology scores for USP9x protein staining of matched normal and tumor samples. Individual tissue spots were examined by an experienced pathologist to confirm the tissue spot identity and visually assigned a score of − (no staining),+ (weak staining of <10% of tissue), ++ (weak staining of 10–25% of tissue), +++ (weak to moderate staining of up to 50% tissue), ++++ (moderate to strong staining of 50–75% of tissue), +++++ (moderate to strong staining of >75% of tissue). In the majority of normal samples and tumors, USP9x staining was localized near the cell membrane, with little or no cytoplasmic staining. However, a small number of tumors showed predominantly cytoplasmic USPx. ccRCC, renal clear cell carcinoma; TCGA, the Cancer Genome atlas.

To examine USP9x levels in other cancers, RNA sequencing data and patient clinical information were downloaded from the TCGA Data Coordination Center. USP9x transcript levels were significantly lower in several cancers compared with normal controls, including breast, thyroid, prostate, liver hepatocellular carcinoma, kidney papillary and ccRCC (Figure 5a). Among these, disease-free survival was significantly worse for ccRCCs patients with bottom quartile USP9x expression compared with those with median or top quartile USP9x expression (KIRC, Figure 5b and Supplementary Figure S6).

There was no significant difference in the levels of USP9x transcripts in ccRCC tumor samples comparing metastatic and non-metastatic disease or comparing low- vs high-grade tumors, but USP9x transcript levels were slightly lower in advanced vs early stage samples in the KIRC data set (Supplementary Figure S7). The VHL and PBRM1 genes are among the most frequently mutated in ccRCC[35]. There was no significant difference in the level of USP9x transcripts in samples separated by the mutational status of the Von Hippel–Lindau tumor suppressor gene VHL, but there was a small decrease in tumors mutant for PBRM1, which encodes the chromatin regulator Polybromo-1 (Supplementary Figure S7). This may reflect effects on the global transcriptome of these tumors.

As USP9x is involved in regulation of protein degradation, we examined USP9x protein levels in ccRCC samples paired with normal kidney tissue from the same patient. Immunohistochemical labeling was performed on tissue arrays containing matched pairs of non-metastatic ccRCC tumors and normal tissue (Supplementary Figure S8). Histological assessment showed moderate to strong expression of USP9x in the normal tissues and lower levels in their matched tumors (Figure 5c and Supplementary Figure S8). This difference was statistically significant using the Wilcoxon matched pairs signed rank test (*P*<0.0001).

## Discussion

**Figure 6:**
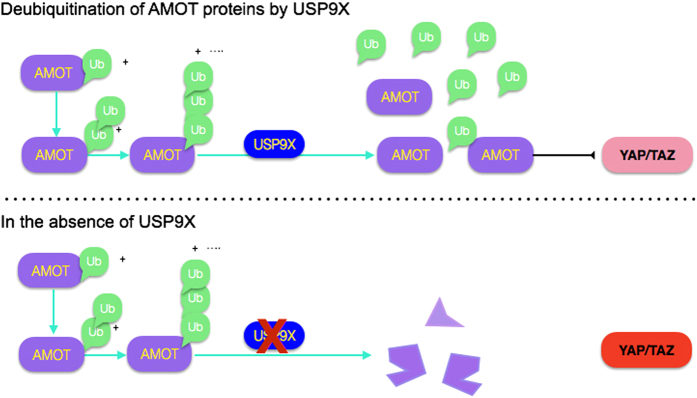
Schematic representation of USP9x activity. USP9x removes ubiquitin (Ub, green) from AMOT family proteins (purple), increasing their stability. AMOT can then limit YAP/TAZ activity. In the absence of USP9x, AMOT is degraded more efficiently and cannot limit YAP/TAZ activity (higher activity depicted in red shading).

YAP is a potent oncogene that drives cancer progression through the upregulation of a number of genes that promote cell growth and inhibition of apoptosis. Two distinct mechanisms for regulation of YAP by ubiquitin-mediated degradation have been reported [8, 36, 37]. The LATS kinases phosphorylate YAP and target it for proteasome-mediated degradation. YAP activity is also controlled by interaction with AMOT, which can limit YAP’s ability to function as a transcriptional cofactor. Here we report that the USP9x deubiquitylase regulates YAP activity by acting on ubiquitylation of the YAP inhibitor, p130AMOT. We demonstrate that loss of USP9x leads to increased ubiquitylation of AMOT, resulting in reduced AMOT levels. Lowering AMOT levels increases YAP/TAZ activity (summarized in Figure 6).

The level of AMOT activity will depend on the balance of the E3 ligases that add ubiquitin and the DUBs that remove it. Several members of the NEDD4 family of E3 ligases, including ITCH have been reported to ubiquitylate AMOT [23]. We have provided evidence that USP9x can act directly on AMOT via residue K496. USP9x has also been reported to remove ubiquitin from the ITCH and SMURF1 ubiquitin ligases, thereby protecting them from degradation [38, 39]. Therefore reduced USP9x levels should lead to lower E3 ligase activity, which would thereby reduce the capacity to ubiquitylate AMOT. However, we observed a net increase in AMOT ubiquitylation in USP9x-depleted cells (Figure 2). Thus, it appears that the direct effect of limiting deubiquitylation of AMOT by USP9x offsets the reduced potential for ubiquitylation due to lower E3 ligase levels.

While our manuscript was in preparation, another study provided evidence that USP9x can work via regulation of AMOTL2 to regulate LATS-mediated phosphorylation of YAP [40]. Our findings differ from those of Kim *et al.* [40] in two respects: (1) We observed that no change in YAP phosphorylation status on USP9x depletion (Figure 2a and b), whereas Kim *et al.* [40] report changes in LATS-mediated phosphorylation of YAP. Choice of cells or experimental design might explain this difference: we examined endogenous YAP/TAZ, whereas YAP and its cofactor TEAD were overexpressed in the experiments reported by Kim *et al* [40]. YAP overexpression triggers feedback regulation via regulation of LATS expression and activity [41–43], as well as through upregulation of AMOTL2 [42]. Changing the balance of regulation in this pathway might influence how stabilization of AMOT proteins affects YAP activity. For example, increased activity of LATS kinases as a result of feedback regulation might promote phosphorylation and stabilization of AMOT proteins. Stabilized AMOT, in turn, can act as a scaffold to promote phosphorylation of YAP by LATS kinases [44–46]. Thus, the basal level of YAP/TAZ could in principle influence the outcome of USP9x-AMOT regulation. (2) Kim *et al.* [40] report increased monoubiquitylation of AMOTL2 on lysine 437 as a consequence of USP9x depletion. Lysine 437 corresponds to Lysine 496 in AMOT (Supplementary Figure S4). In the course of our experiments, we also observed monoubiquitylation of AMOT, when AMOT was strongly overexpressed (Supplementary Figure S9). This contrasted with the effects of USP9x on cells with moderate AMOT levels, in which AMOT was polyubiquitylated and degraded in response to USP9x depletion (Figures 3c and 4d). We suggest that monoubiquitylation may be a consequence of saturating the capacity of the cells to ubiquitylate AMOT. Further work will be needed to explore the differences between the mechanisms reported in these two studies.

### USP9x in cancer

In addition to targeting the Hippo pathway via regulation of AMOT, several other cancer-relevant targets have been reported for the USP9x deubiquitylase. USP9x has been reported to increase SMAD4 activity by removing an inhibitory ubiquitin moiety [47]. Thus loss of USP9x can increase TGFβ signaling, potentially contributing to tumorigenesis. USP9x activity has also been linked to stress-induced activation of the JNK pathway through stabilization of ASK1, a member of the MAPKKK family [48]. In this scenario, low USP9x levels would lead to reduced JNK activation and to reduced stress-induced apoptosis. Reduced sensitivity to oxidative stress could be another mechanism by which low USP9x levels contribute to disease progression.

In contrast to solid tumors of epithelial origin, hematological tumors consistently show reduced YAP expression, which protects cells from DNA-damage induced apoptosis [49]. Interestingly, USP9x has been reported to have tumor-promoting activity, acting via stabilization of the pro-survival protein MCL1 in hematological tumors [33]. In this context it is noteworthy that the effects of USP9x on stability of the E3 ligase ITCH were more potent in PDA cells grown in suspension, than in substratum attached cells [20]. In PDA cells, USP9x depletion had no effect on ITCH targets known to be involved in cell survival [20]. It is therefore tempting to speculate that changes in USP9x activity might have a more significant effect on ITCH activity in lymphoma. Possible roles of USP9x-mediated regulation of AMOT in lymphoma remain to be explored. It seems likely that the relationship between USP9x levels and clinical outcome in different cancers will reflect the balance of its activity on multiple pathways.

## Materials and Methods

### Reagents

Antibodies to phospho-YAP Ser127 (#4911), YAP (#4912), YAP/TAZ (#8418), phospho-MST1/2 (#3681), MST1 (#3682), MST2 (#3952), LATS1 (#9153), LATS2 (#13646), pLATS1/2 Tr1079/1041 (#9159) and Myc Tag (#2278) were from Cell Signaling Technology, Danvers, MA, USA. Antibodies to HA (#sc-7392) and NF2 (#sc-332) were from Santa Cruz Biotechnology, Dallas, TX, USA. Anti-AMOT was from ABNOVA (Taipei City, China) Anti-AMOTL1 and anti-Actin were from Sigma (Sigma-Aldrich, St Louis, MO, USA). USPx and V5 antibodies were from Bethyl Laboratories Inc., Montgomery, TX, USA. Anti-HA, anti-V5 and Flag-conjugated agarose beads were from Sigma. WP130 was obtained from Selleck chemicals, Houston, TX, USA (#S2243).

### Plasmids and cell culture

The shRNA library consists of 116 pools of 4 non-overlapping shRNAs targeting known or putative human DUBs as described [19] supplemented with 22 additional shRNAs targeting newly identified DUBs (details available on request). pBabe HA-LATS2, AMOT, AMOTL1 and AMOTL2 expression plasmids were from SW Chan (IMCB, Singapore). Mutant versions of AMOT were made by PCR. 8×GTIIC-luciferase was obtained from Addgene (Cambridge, MA, USA plasmid # 34615). The pRL-CMV was purchased from Promega, Madison, WI, USA (Renilla, #E2261). The Myc-tagged ubiquitin expression vector was a gift from Hong Yi (IMCB). 480SBY vector was generated by replacing the Blast ORF of pRetrosuper-Blast [50] with a Blast-YFP fusion. LATS2 and USPx shRNAs were cloned into the 480SBY vector. LATS2 shRNA was described in reference 50. All constructs were verified by DNA sequencing. Dharmacon ON-TARGETplus USPx siRNA was obtained from GE Healthcare, Park BrÃ¸ndby, Denmark. U2OS, HEK293 and HEK293T cells were obtained from ATCC, Wesel, Germany and cultured in DMEM (Invitrogen, Naerum, Denmark) with 10% fetal calf serum (HyClone) and 1% penicillin-streptomycin.

### Luciferase assays

HEK 293 T cells were seeded in 48-well plates (37 500 cells per well) 24 h before transfection with 2 ng of pRL-CMV, 125 ng of 8×-GTIIC TEAD reporter and 250 ng of individual pools of DUB shRNAs per well, using the calcium-phosphate method. Luciferase activity was measured 48 h after transfection using a dual luciferase kit (Promega E1960). Firefly luciferase activity was normalized to Renilla activity and the ratio of firefly/Renilla was normalized to the empty vector controls. Assays were replicated with at least three independent transfections.

### Viral transduction and soft agar assays

Amphotropic retroviruses were made as described previously [51]. Supernatant from transfected Phoenix-Ampho cells (NORDIC BIOSITE APS, Copenhagen, Denmark) was harvested at 36–48 h and frozen in aliquots. Cells were plated to reach 70% confluence when infected with virus overnight in the presence of 8 μg ml^−1^ polybrene. Antibiotic selection was started at 36 h. Stable cells were assessed for soft agar growth, as described [10].

### Quantitative real-time RT–PCR

RNA was extracted in TriZol (Invitrogen) and cDNA was made using iScript (Bio-Rad Laboratories Inc., Hercules, CA, USA) with random hexamer primers. At least two pairs of qPCR primers were tested for specificity and sensitivity for each mRNA. Real-time PCR used SyberGreen I Mix on a QuantStudio 6 Flex machine (ABI, Thermo Fisher Scientific, Waltham, MA, USA). Primers are in Supplementary Table S2].

### Immunoprecipitation

Transfected HEK293T cells were washed once with cold TBS (20 mm Tris-HCl pH 7.5, 150 mm NaCl) and lysed with buffer containing 50 mm HEPES pH 7.5, 150 mm NaCl, 5% Glycerol, 0.5% Triton X-100, 1.5 mm MgCl2, 1 mm EGTA supplemented with 20 μg ml^−1^ RNAase A, 1 mm DTT, 1 mm Na_3_VO_4_ and protease inhibitor cocktail (Roche, Basel, Switzerland). Cell lysates were immunoprecipitated for 2 h at 4 °C with anti-HA agarose (#A2095, Sigma). Agarose beads were washed 4× with lysis buffer and eluted as recommended by the manufacturer.

### Immunoblotting and ubiquitylation assays

For total protein analysis and interaction studies, transfected HEK 293 T cells were washed once with cold TBS and lysed with phospholipase C buffer (Wells:2006ic) containing 50 mm HEPES pH 7.5, 150 mm NaCl, 5% Glycerol, 0.5% Triton X-100, 1.5 mm MgCl2, 1 mm EGTA supplemented with 20 μg ml^−1^ RNAase A, 1 mm DTT, 1 mm Na3VO4 and protease inhibitor cocktail. Co-IP was performed using anti-HA agarose for 2 h at 4C on a rotator. Agarose bead was washed four times with lysis buffer and immune complexes were eluted as recommended by the manufacturer. Immune complexes were detected with western Lightning Plus-ECL reagent (Perkin Elmer, Waltham, MA, USA).

For ubiquitin assay and mass spectrometry (MS) detection of the ubiquitylation of AMOT, HEK293 and HEK293T cells were co-transfected to express Myc-tagged ubiquitin (2 μg per 10-cm dish), HA-tagged AMOT wild-type or mutant plasmids (5 μg) and USPx shRNA (10 μg) or control vector as indicated using the calcium-phosphate method. Forty eight hours later, cells were washed with cold TBS and lysed in modified RIPA buffer containing 25 mm Tris/HCl, pH 7.5, 150 mm NaCl, 1% Nonidet NP40, 0.1% Na-deoxycholate, 1 mm EDTA, 5 mm NaF, 5 mm β-glycerophosphate, 1 mm Na_3_VO_4_, freshly complemented with 10 mm NEM and protease inhibitor cocktail. HA-AMOT was immunoprecipitated using anti-HA agarose. Agarose bead was washed four times with lysis buffer and complexes were eluted and subjected for detection of ubiquitylated AMOT by western blotting or ubiquitylated lysine by MS.

### Sample preparation and MS

HEK cells were SILAC-labeled in medium containing l-arginine and l-lysine or l-arginine-U-^13^C_6_-^15^N_4_ and l-lysine-U-^13^C_6_-^15^N_2_ (Cambridge Isotope Laboratories, Tewksbury, MA, USA) as previously described [52]. Peptides were analyzed on a quadrupole Orbitrap mass spectrometer (Q-Exactive plus, Thermo Fisher Scientific, Waltham, MA, USA) equipped with a nanoflow HPLC system (Thermo Scientific), as previously described [53]. Peptides were loaded onto C18 reversed phase columns (15 cm length, 75 μm inner diameter) and eluted with a linear gradient from 8–40% acetonitrile containing 0.5% acetic acid. The mass spectrometer was operated in a data-dependent mode, automatically switching between MS and MS/MS acquisition. Survey full scan MS spectra (*m/z* 300–1 750) were acquired in the Orbitrap. The 10 most intense ions were sequentially isolated and fragmented by higher-energy C-trap dissociation (HCD [54). An ion selection threshold of 50 000 counts was used. Peptides with unassigned charge states, as well as with charge state less than +2 were excluded from fragmentation. Fragment spectra were acquired in the Orbitrap mass analyzer (Thermo Fisher Scientific).

### Peptide identification and data analysis

Raw data were analyzed using MaxQuant (version 1.4.0.03, [55]). A Uniprot database against human proteome obtained from the UniProtKB (February 2012 release) was taken to search for parent ion and MS/MS spectra using Andromeda search engine [56]. Spectra were searched with a mass tolerance of 6 p.p.m. in MS mode, 20 p.p.m. in HCD MS2 mode, strict trypsin specificity and allowing maximally two missed cleavage sites. Cysteine carbamidomethylation was searched as a fixed modification, whereas N-terminal protein acetylation, methionine oxidation and *n*-ethylmaleimide modification of cysteines and di-glycine-lysine were searched as variable modifications. Site localization probabilities were determined by MaxQuant using the PTM scoring algorithm [55, 57]. The data set was filtered based on posterior error probability to arrive at a false discovery rate of <0.01 estimated using a target-decoy approach [58].

### Immunohistochemical analysis

Human kidney normal-tumor paired tissue arrays KD1503 and KD1504 representing tumors in duplicates from 98 kidney clear cell carcinoma and their matched normal tissues were obtained from US Biomax, Rockville, MD, USA. Immunostaining of tissue arrays was performed as described [59]. Sections were dewaxed in xylene and rehydrated; antigen retrieval was performed by boiling the slides in 10 mm citrate buffer pH 6.0 for 20 min after. Endogenous peroxidase activity was blocked by treatment with 3% hydrogen peroxide in methanol for 15 min. After four washes of Tris buffer saline (20 mm Tris, 200 mm NaCl, pH 7.6), the sections were pre-incubated with Ultra V Block solution (Thermo scientific) for 10 min and incubated for 2 h with USPx antibody. Immunohistochemistry was performed using the streptavidin–biotin peroxidase complex method according to the manufacturer’s instructions (UltraVision HRP DAB system, Thermo scientific) using DAB as the chromogen. Individual tissue spots were examined by an experienced pathologist to confirm the tissue spot identity and visually assigned a score of 0 (no staining), 1 (weak staining of <10% of tissue), 2 (weak staining of 10–25% of tissue), 3 (weak to moderate staining of up to 50% tissue), 4 (moderate to strong staining of 50–75% of tissue) and 5 (moderate to strong staining of >75% of tissue).

### Gene expression and survival analysis in cancer patient data

Publicly available RNA sequencing data and patient clinical information was downloaded from TCGA Data Coordination Center (DCC) data server and assembled using TCGA assembler version 1.0.3. on 27 February 2015 [60]. Gene expression values were normalized to a fixed upper quartile value of 1 000 using RSEM (RNA-Seq by Expectation maximization). Statistical analysis was done in R software (http://www.r-project.org). Statistical significance was determined using Mann–Whitney tests. To calculate comparisons between multiple groups, pairwise Wilcoxon tests with Bonferroni corrections for multiple testing were applied. For progression free survival analysis, new tumor event diagnostic indicator or death was used as an event. For this analysis, gene expression was split into groups by upper and lower quartile. Cox proportional hazards regression model was used to calculate *P*-values. TCGA KIRC gene level non-silent somatic mutation data (broad automated) was downloaded from UCSC Cancer Genome Browser website (https://genome-cancer.ucsc.edu) on 24 February 2015.
