# Supplementary material for: Deubiquitylating enzyme USP9x regulates hippo pathway activity by controlling angiomotin protein turnover
Source: Cell Discov. 2016 Mar 29;2:16001–. doi: 10.1038/celldisc.2016.1 (PMC4849470; doi:10.1038/celldisc.2016.1)
Supplement: Supplementary Figure S1 [file celldisc20161-s1.pdf]

Figure S1. USP9x depletion increases soft agar colony formation by HEK293T cells.

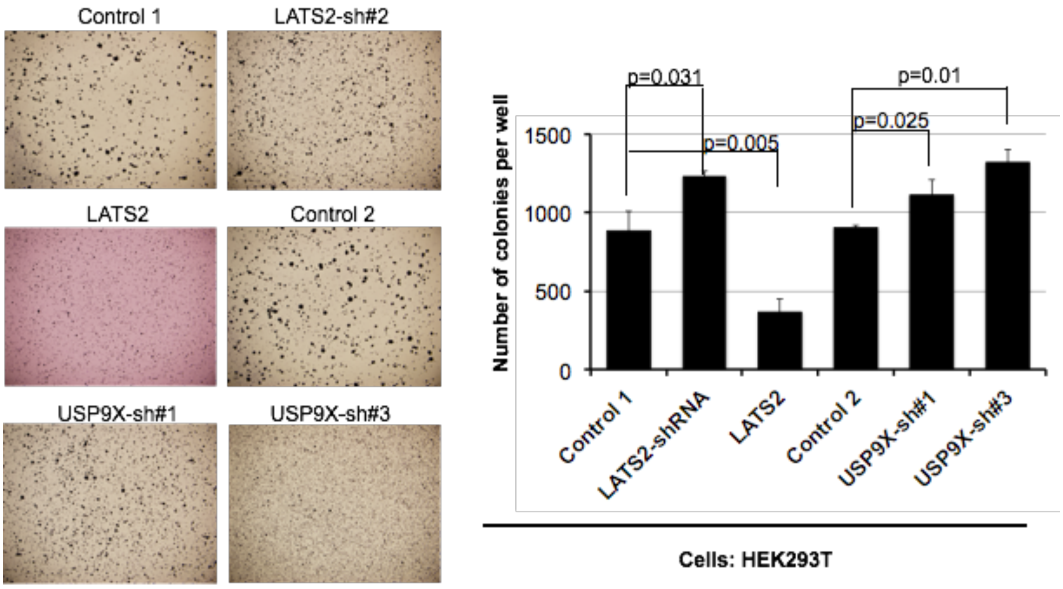

HEK293T cells were retrovirally transduced to express shRNAs targeting USP9x, LATS2 or control shRNA. After selection for expression of the vector cells were plated on soft agar. Colonies were stained with MTT after 8 days and counted (representative images shown at left). Right: counts of colony number per well from 3 independent experiments. Error bars: mean ± SD. P values shown for pairwise combinations by t-test (two-tailed, unequal variance).
