# Supplementary material for: Deubiquitylating enzyme USP9x regulates hippo pathway activity by controlling angiomotin protein turnover
Source: Cell Discov. 2016 Mar 29;2:16001–. doi: 10.1038/celldisc.2016.1 (PMC4849470; doi:10.1038/celldisc.2016.1)
Supplement: Supplementary Figure S2 [file celldisc20161-s2.pdf]

Figure S2. Additional co-IP data

a

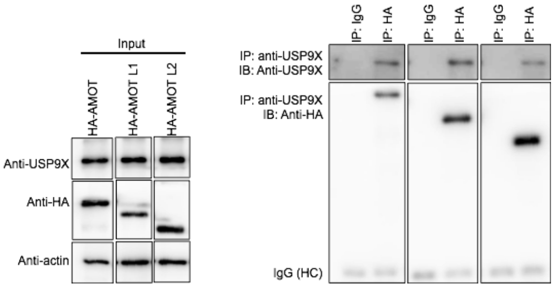

Co-IP of AMOT family members with USP9x. HEK293T cells were transfected to express HA-tagged versions of p-130 MOT, AMOT-L1 or AMOT-L2. Cell lysates were immunoprecipitated with anti-USP9x, or with IgG control. Blots were probed with anti-HA to visualize the AMOT family members. Anti-actin controls for loading in the input lysates. Cross reaction of the secondary antibody with the IgG heavy chain controls for the loading of the IP.

b

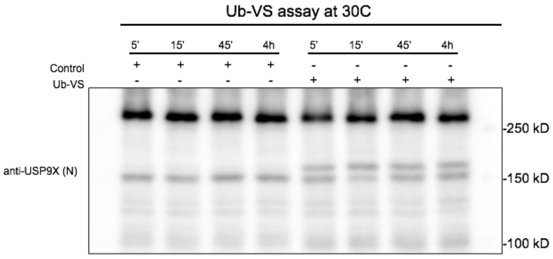

Cleavage of USP1 is associated with activation of the enzyme (Cohn et al 2007 Mol Cell 28:786-797). Intact and cleaved forms of USP9x can be detected by antibody to the N-terminus of the protein. The cleaved form is catalytically active, as shown by incorporation of Ub-VS, which shifts mobility to a slower form (as in Cohn et al 2007). Antibody to the C-terminus only detects the full length inactive protein (not shown).
