# Supplementary material for: Deubiquitylating enzyme USP9x regulates hippo pathway activity by controlling angiomotin protein turnover
Source: Cell Discov. 2016 Mar 29;2:16001–. doi: 10.1038/celldisc.2016.1 (PMC4849470; doi:10.1038/celldisc.2016.1)
Supplement: Supplementary Figure S3 [file celldisc20161-s3.pdf]

Figure S3. USP9x affects AMOT protein stability

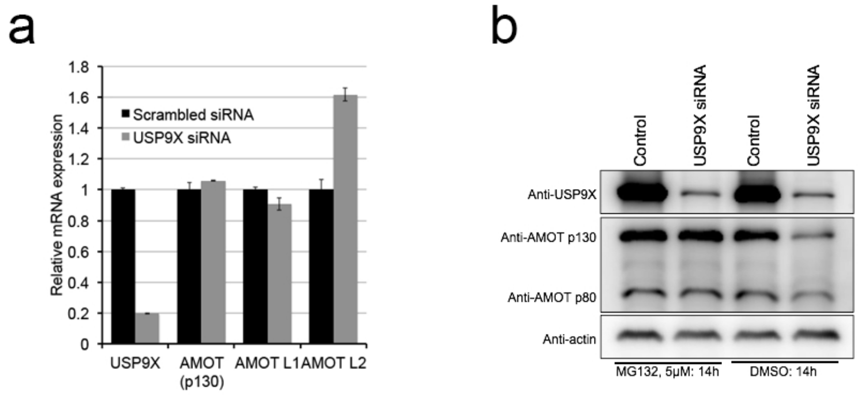

(a) AMOT family mRNA levels measured by quantitative RT-PCR. Note that depletion of USP9x (left columns) did not reduce the level of p130-AMOT, AMOTL1 or AMOTL2 mRNAs. Reduction of the corresponding protein levels from this experiment are shown in figure 2f and g. Note that the level of AMOTL2 transcript increased, consistent with known effects of YAP activity on AMOTL2 expression. Despite the increase in AMOTL2 mRNA, depletion of USP9x led to a reduced level of AMOTL2 (see figure 2f).

(b) Effect of blocking proteasome activity on regulation of AMOT levels by USP9x. Cells were treated with shRNA to deplete USP9x (upper blot), and treated with MG132 or DMSO vehicle control to block proteasome activity as indicated in the bottom panel. Note that the decrease in p130-AMOT in USP9x depleted cells was blocked by MG132 treatment. This suggests that the effect of USP9x depletion is due to proteasome mediated degradation of AMOT protein.
