# Supplementary material for: Deubiquitylating enzyme USP9x regulates hippo pathway activity by controlling angiomotin protein turnover
Source: Cell Discov. 2016 Mar 29;2:16001–. doi: 10.1038/celldisc.2016.1 (PMC4849470; doi:10.1038/celldisc.2016.1)
Supplement: Supplementary Figure S4 [file celldisc20161-s4.pdf]

Figure S4. Alignment of AMOT family proteins

```
                              496
AMOT:     … NLVKSSSKREALEKAMRNKLEGEIRRM …
                              503
AMOT L1:  … SLVKSTTKRESLDKAMRNKLEGEIRRL …
                              437
AMOT L2:  … SLTRASSKREALEKTMRNKMDSEMRRL …
```

The Lysine residue corresponding to position K496 in AMOT is conserved among the AMOT-like family members (residue location in each protein indicated in red). AMOTL1 and AMOT also share a Lysine at the position corresponding to K481 in AMOT. In AMOTL2 this residue is an Arginine.
