# Supplementary material for: Deubiquitylating enzyme USP9x regulates hippo pathway activity by controlling angiomotin protein turnover
Source: Cell Discov. 2016 Mar 29;2:16001–. doi: 10.1038/celldisc.2016.1 (PMC4849470; doi:10.1038/celldisc.2016.1)
Supplement: Supplementary Figure S5 [file celldisc20161-s5.pdf]

Figure S5. Mutant forms of AMOT

(a)

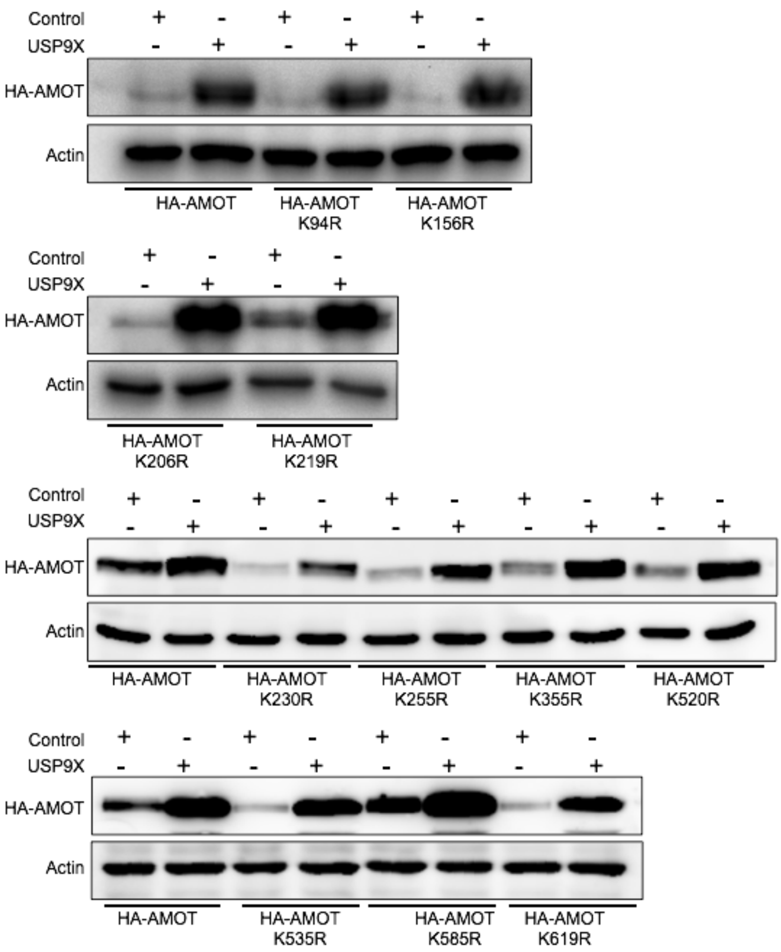

(a) Effect of USP9x on mutant forms of AMOT, replacing lysine residues predicted to be ubiquitylated (http://www.phosphosite.org/). HEK293T cells were transfected to express moderate levels of HA-tagged AMOT carrying the mutations converting the indicated Lysine residues to Arginine. Blots were probed with anti-HA to visualize AMOT and anti-actin as a loading control. We have not explored the effect on some mutations on base-line AMOT expression. None of the mutations prevented stabilization of AMOT in cells expressing USP9x.

(b)

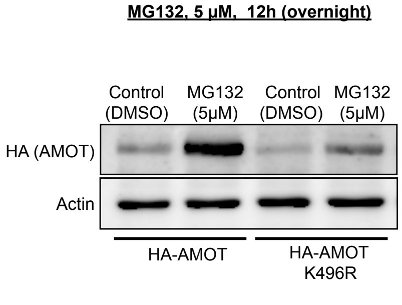

Inhibition of the proteasome with MG132 stabilized native AMOT, but had much less effect on AMOT K496R, suggesting that the mutant protein is less sensitive to proteasome mediated degradation.
