# Supplementary material for: Deubiquitylating enzyme USP9x regulates hippo pathway activity by controlling angiomotin protein turnover
Source: Cell Discov. 2016 Mar 29;2:16001–. doi: 10.1038/celldisc.2016.1 (PMC4849470; doi:10.1038/celldisc.2016.1)
Supplement: Supplementary Figure S6 [file celldisc20161-s6.pdf]

Figure S6. Disease free survival

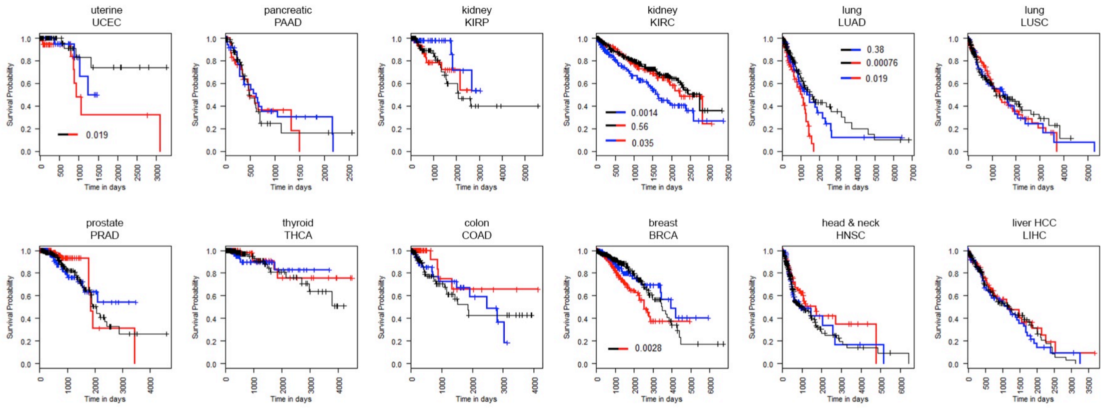

Plots of disease free survival for patients from the indicated TCGA data sets. Patients were grouped by the level of USP9x expression: blue = bottom quartile; black = middle 50%; red = top quartile of USP9x expression. *P*-values are indicated for the samples with significant differences between the low and median or high and median expression groups. Among these cancers only kidney renal clear cell carcinoma (KIRC) showed a significantly worse survival for the patients with low USP9x expression.
