# Supplementary material for: Deubiquitylating enzyme USP9x regulates hippo pathway activity by controlling angiomotin protein turnover
Source: Cell Discov. 2016 Mar 29;2:16001–. doi: 10.1038/celldisc.2016.1 (PMC4849470; doi:10.1038/celldisc.2016.1)
Supplement: Supplementary Figure S7 [file celldisc20161-s7.pdf]

**Figure S7. USP9x transcript levels in ccRCC**

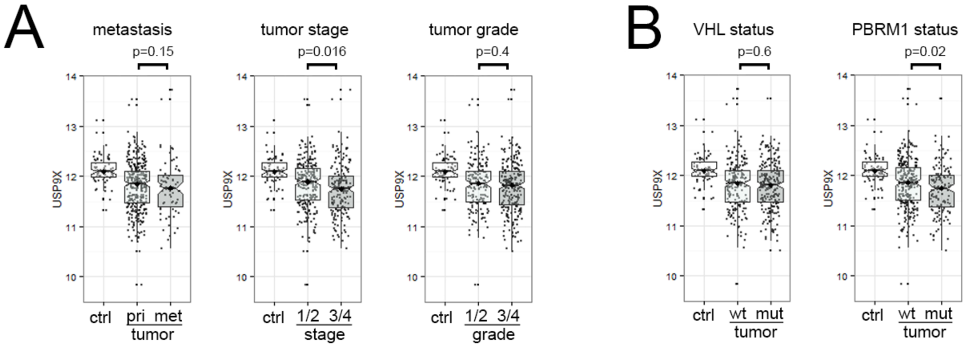


(A) USP9x expression in TCGA KIRC (ccRCC) patients grouped by disease features. From left: metastatic vs non-metastatic (pri); stage 1+2 vs stage 3+4 (groups were merged to allow for increase sample number to test significance); grade 1+2 vs grade 3+4. There was a small difference between the stage1+2 and stage 3+4 tumors.

(B) TCGA KIRC patients grouped by mutational status for VHL and PBRM1. There was a small magnitude, but statistically significant difference with PBRM1 status.
