# Supplementary material for: Deubiquitylating enzyme USP9x regulates hippo pathway activity by controlling angiomotin protein turnover
Source: Cell Discov. 2016 Mar 29;2:16001–. doi: 10.1038/celldisc.2016.1 (PMC4849470; doi:10.1038/celldisc.2016.1)
Supplement: Supplementary Figure S8 [file celldisc20161-s8.pdf]

Figure S8. USP9x protein levels in matched pairs of ccRCC and normal kidney tissue

(a) Immunohistochemical labeling with anti-USP9x on kidney cancer tissue microarrays.

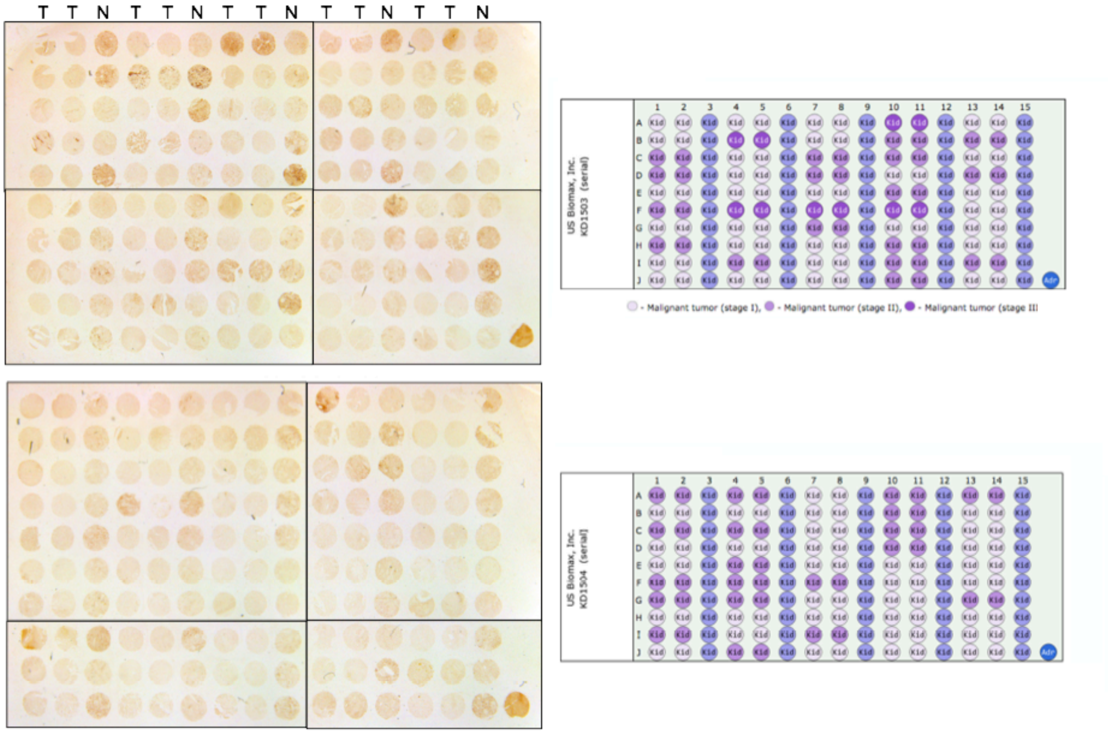

Upper panel: slide KD1503. Lower panel slide KD1504. At right: manufacturers code for tumor stage.

(b)

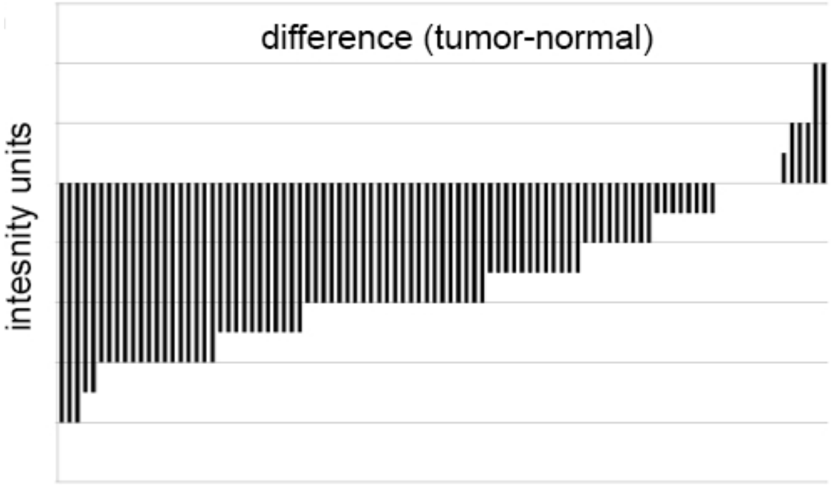

Plot showing the difference in USP9x intensity scores between the tumors and their matched normal tissue. The score for the normal tissue was subtracted from the average score of the two tumor spots for each individual. Scoring as in Fig 4c, represented as numerical values.
