# Supplementary material for: Deubiquitylating enzyme USP9x regulates hippo pathway activity by controlling angiomotin protein turnover
Source: Cell Discov. 2016 Mar 29;2:16001–. doi: 10.1038/celldisc.2016.1 (PMC4849470; doi:10.1038/celldisc.2016.1)
Supplement: Supplementary Figure S9 [file celldisc20161-s9.pdf]

Figure S9: effects of AMOT overexpression

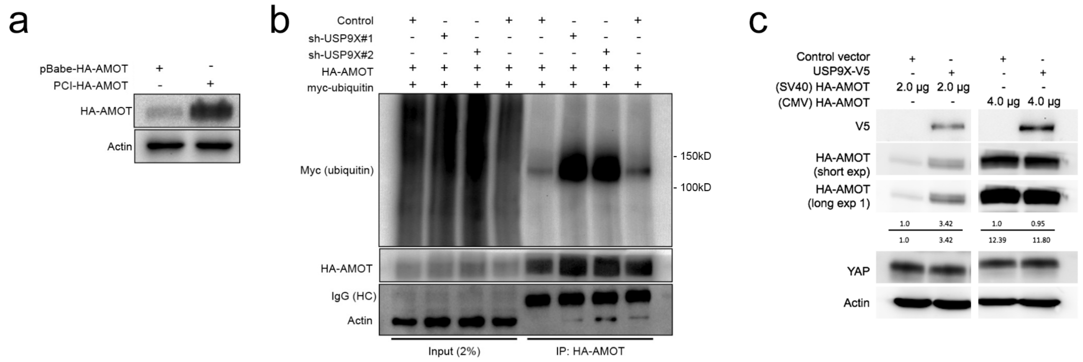

(a) Comparison of the levels of AMOT protein produced by pBabe and PCI expression vectors. 10μg of DNA were transfected per 10cm dish for each vector.

(b) Ubiquitylation assay performed on cells transfected with PCI-HA-AMOT to express high levels of AMOT protein. Under these conditions depletion of USP9x led to accumulation of a monoubiquitylated form of AMOT. This differs from the result obtained when AMOT was expressed at lower levels using pBabe vectors, as shown in figures 3c and 4d.

(c) Comparison of the effects of USP9x overexpression in cells expressing moderate and high levels of HA-AMOT in HEK293T cells. Cells were harvested 48h after transfection. USP9x expression led to an increase in AMOT protein expressed at moderate levels but did not affect the amount of AMOT in cells overexpressing the protein at higher levels. Quantification of the immunoblots was done using a phosphorimager in the linear exposure range. YAP and Actin levels are shown as controls.

Kim et al (ref 39) recently reported mono-ubiquitylation of AMOTL2 after USP9x depletion and no effect of USP9x expression on AMOTL2 levels. We suggest that the difference between their results and those obtained her could be due to the level of AMOT/AMOTL2 expression used in the two studies.
