# Supplementary material for: Deubiquitylating enzyme USP9x regulates hippo pathway activity by controlling angiomotin protein turnover
Source: Cell Discov. 2016 Mar 29;2:16001–. doi: 10.1038/celldisc.2016.1 (PMC4849470; doi:10.1038/celldisc.2016.1)
Supplement: Supplementary Table S1 [file celldisc20161-s10.pdf]

Table S1. Relative YAP/TAZ activity

	Control and DUB shRNA pools	DUB name	Ensembl Gene ID	NCBI and others	1st batch	2nd batch	3rd batch
1	Control 1				1	1	1
2	Control 2				1.64	2.51	0.47
3	LATS2 shRNA				9.67	9.16	11.42
4	LATS2				0.09	0.16	0.03
5	DUB1	ISOT-3	ENSG00000058056		1.27	1.27	1.49
6	DUB2	USP12	ENSG00000152484		1.03	1.65	1.48
7	DUB3	USP11	ENSG00000102226		1.76	2.16	1.53
8	DUB4	USP10	ENSG00000103194		1.14	1.27	1.57
9	DUB5	HAUSP	ENSG00000171616		0.85	0.96	1.18
10	DUB6	USP8	ENSG00000138592		1.68	1.64	1.21
11	DUB7	USP40	ENSG00000085982		0.87	0.76	1.36
12	DUB8	USP35	ENSG00000118369		0.98	0.91	1.15
13	DUB9	USP52	ENSG00000135473		0.99	0.91	1.40
14	DUB10	USP46		NM_022832	1.66	1.23	1.29
15	DUB11	USP31	ENSG00000103404		1.07	1.16	1.88
16	DUB12	USP29 , pool 1	ENSG00000131864		1.59	1.53	2.05
17	DUB13	USP2	ENSG00000036672		0.29	0.69	0.29
18	DUB14	USP31	ENSG00000090686	NM_032236	2.27	0.91	1.75
19	DUB15	USP30	ENSG00000135093		3.52	2.06	1.40
20	DUB16	UCHL5	ENSG00000116750		1.02	1.66	1.40
21	DUB17	UCHL3	ENSG00000118939		0.98	1.08	1.57
22	DUB18	UCHL1	ENSG00000154277		3.00	2.96	2.32
23	DUB19	USP14	ENSG00000101557		1.70	1.91	2.15
24	DUB20	USP3, pool 1	ENSG00000140455		1.05	0.65	1.48
25	DUB21	USP38		AF211481	0.45	0.43	0.25
26	DUB22	USP23	ENSG00000143258		0.45	0.69	0.49
27	DUB23	USP44	ENSG00000136014		1.11	0.64	0.85
28	DUB24	USP39	ENSG00000168883		0.95	1.66	0.91
29	DUB25	XM_089437	XM_089437		0.42	0.56	0.43
30	DUB26	USP36	ENSG00000055483		0.68	0.85	0.85
31	DUB27	UBP-32.7 , pool 1	ENSG00000123552		0.58	0.72	0.92
32	DUB28	USP4	ENSG00000114316		1.63	1.12	1.10
33	DUB29	USP27 homolog , pool 1		XM_093149	1.22	0.92	1.54
34	DUB30	FLJ12697	ENSG00000106346		0.86	0.75	0.70
35	DUB31	BRCA1 ASSOCIATED PROTEIN-1, pool 1	ENSG00000163930		2.21	2.90	1.85
36	DUB32	USP15	ENSG00000135655		0.44	0.53	0.82
37	DUB33	Homo sapiens similar to IL-2 DUB, pool 1		XM_302441	0.58	1.07	1.07
38	DUB34	USP19, pool 1		XM_114325	3.14	2.16	1.93
39	DUB35	USP18	ENSG00000099996		0.41	0.84	1.51
40	DUB36	CYLD	ENSG00000083799		0.93	1.24	0.95
41	DUB37	USP20	ENSG00000136878		1.53	1.42	1.25
42	DUB38	USP24	ENSG00000162402		1.10	0.60	1.49
43	DUB39	KIAA1594	ENSG00000135913		0.93	0.66	0.94
44	DUB40	USP47		NM_017944	0.82	0.74	1.25
45	DUB41	USP53		XM_052597	1.88	3.40	1.48
46	DUB42	USP25	ENSG00000155313		1.08	1.49	1.12
47	DUB43	UBP16'	ENSG00000152263		1.61	1.06	1.59
48	DUB44	USP16	ENSG00000156256		1.55	0.68	1.34
49	DUB45	USP9X pool 1	ENSG00000124486		0.67	1.18	0.68
50	DUB46	USP9Y	ENSG00000114374		3.43	2.92	1.86
51	DUB47	USP1		BC050525	0.53	0.32	0.25
52	DUB48	USP49 pool 1		BC014176	2.96	1.90	1.27
53	DUB49	UCH5	ENSG00000111667		0.81	1.15	1.17
54	DUB50	KIAA0565		XM_039912	1.24	1.44	1.71
55	DUB51	UCH26	ENSG00000134588		1.48	1.57	1.28
56	DUB52	KIAA1097 (VDUI)	ENSG00000077254		0.96	1.03	1.64
57	DUB53	USP6 (TRE-2)	ENSG00000129204		0.75	0.85	0.66
58	DUB54	USP22	ENSG00000124422		1.25	1.48	1.77
59	DUB55	USP28	ENSG00000048028		0.95	1.13	1.48
60	DUB56	USP29 pool 2	ENSG00000131864		2.74	1.97	1.87
61	DUB57	MOUSE USP27 HOMOLOG , pool 2		XM_093149	1.47	1.23	1.58
62	DUB58	UCH2, pool 2	ENSG00000036672		1.11	2.01	1.66
63	DUB59	UCH3, pool 2	ENSG00000140455		0.29	0.21	0.24
64	DUB60	UCH23, pool 2	ENSG00000143258		5.25	6.22	5.19
65	DUB61	UCH23, pool 3	ENSG00000143258		1.22	0.90	0.67
66	DUB62	UBP-32.7, pool 2	ENSG00000123552		0.78	0.58	0.97
67	DUB63	USP19, pool 2		XM_114325	0.26	0.22	0.27
68	DUB64	USP47, pool 2		NM_017944	0.83	0.54	1.24
69	DUB65	USP49, pool 2		BC014176	0.79	0.55	0.67
70	DUB66	KIAA0565 pool 2		XM_039912	0.62	0.26	0.88
71	DUB67	No description	ENSG00000152263		0.48	0.28	0.86
72	DUB68	Homo sapiens similar to IL-2 DUB, pool 2		XM_302441	0.71	0.38	0.73
73	DUB69	BRCA1 ASSOCIATED PROTEIN-1, pool 2			0.98	0.23	0.33
74	DUB101	Ataxin-3-like	ENSG00000123594		1.41	1.58	0.96
75	DUB102	Ataxin-3	ENSG00000066427		1.58	2.01	0.79
76	DUB103	JOSD3	ENSG00000166012		1.87	3.89	1.25
77	DUB104	HIN-1	ENSG00000164164		0.82	1.16	1.12
78	DUB105	Otubain-1	ENSG00000167770		0.50	0.48	1.14
79	DUB106	Otubain-2	ENSG00000089723		0.78	1.15	0.86
80	DUB107	RPN8	ENSG00000103035		1.21	1.44	1.45
81	DUB108	TL132	ENSG00000188933		1.87	2.43	2.28

82	DUB109	TRABID	ENSG00000019995		0.48	0.59	0.41
83	DUB110	USP42	ENSG00000106346		0.98	1.45	0.72
84	DUB111	USP48	ENSG00000090686		1.27	1.36	0.94
85	DUB112	USP49	ENSG00000164663		0.77	1.00	1.71
86	DUB113	USP54	ENSG00000166348		1.30	2.07	1.88
87	DUB114	VCIP135	ENSG00000175073		1.61	1.76	1.17
88	DUB115	OTUD2, YOD1	ENSG00000180667		1.48	2.26	1.16
89	DUB116	BRCC36, BRCC3	ENSG00000185515		1.51	2.53	1.64
90	DUB117	Cezanne-1	ENSG00000163113		1.07	0.82	1.28
91	DUB118	Cezanne-2	ENSG00000169918		1.28	1.16	1.62
92	DUB119	CSN5, JAB1, SGN5	ENSG00000121022		1.59	1.03	1.60
93	DUB120	CSN6, SGN6	ENSG00000168090		1.09	1.06	1.55
94	DUB121	CSN6, SGN6	ENSG00000168090		1.54	2.17	1.11
95	DUB122	DUB-3	ENSG00000182945		1.64	2.35	1.02
96	DUB123	eIF-epsilon	ENSG00000175390		2.57	3.27	1.86
97	DUB124	eIF-3-gamma	ENSG00000147677		1.85	1.51	2.14
98	DUB125	USP17L17		NM_001256857	3.42	2.74	2.14
99	DUB126	No description	ENSG00000198817		3.65	5.04	3.27
100	DUB127	HIN-1-like	ENSG00000118976		1.11	1.20	1.53
101	DUB128	HIN-6	ENSG00000189401		1.54	1.23	0.95
102	DUB129	IFP38	ENSG00000187684		0.91	0.68	0.83
103	DUB130	JAMM2	ENSG00000162601		1.78	1.71	1.57
104	DUB131	JAMM3	ENSG00000008382		1.01	0.70	1.13
105	DUB132	*JOSD1*	ENSG00000100221		1.29	1.19	0.74
106	DUB133	*JOSD2*	ENSG00000161677		1.14	0.94	1.67
107	DUB134	OTUD1	ENSG00000165312		0.77	1.44	1.26
108	DUB135	OTUD5	ENSG00000068308		1.40	1.43	1.08
109	DUB136	OTUD6B	ENSG00000155100		1.25	0.89	0.54
110	DUB137	POH1, RPN11	ENSG00000115233		1.13	0.85	0.71
111	DUB138	PRP8	ENSG00000174231		1.88	2.48	1.49
112	DUB139	STAMBP, AMSH	ENSG00000124356		0.87	0.89	1.47
113	DUB140	AMSH-like	ENSG00000138134		1.08	0.61	0.80
114	DUB141	TL132-like	ENSG00000189423		1.68	2.38	0.86
115	DUB142	USP11		NM_004651	2.59	3.27	1.40
116	DUB143	USP4		NM_003363	2.09	3.11	1.62
117	DUB144	USP37		NM_020935	1.54	1.68	1.12
118	DUB145	USP9X pool 2		NM_001039590	6.64	4.29	5.76
119	DUB146	USP9X pool 3		NM_001039591	9.57	14.61	7.77
120	DUB147	OTUD4		NM_001102653	0.85	0.72	0.70
