# Supplementary material for: Deubiquitylating enzyme USP9x regulates hippo pathway activity by controlling angiomotin protein turnover
Source: Cell Discov. 2016 Mar 29;2:16001–. doi: 10.1038/celldisc.2016.1 (PMC4849470; doi:10.1038/celldisc.2016.1)
Supplement: Supplementary Table S2 [file celldisc20161-s11.pdf]

Table S2. shRNA target sequences and primers used for qPCR.

**shRNA sequences**	
USP9X	sh1 target sequence: AAGATGAGGAACCTGCATTTC
	sh2 target sequence: AGCAGTGAGTGGCTGGAAGTT
	sh3 target sequence: GTTAGAGAAGATTCTTCGTTT
USP9X siRNA (smart pool)	AGAAAUCGCUGGUAUAAAU<br>ACACGAUGCUUUAGAAAUUU<br>GUACGACGAUGUAUUCUCA<br>GAAAUAACUUCCUACCGAA
**PCR primers**	
LATS2	Forward:5'-TTGCTGATGTACTCCAGGGC-3'
	Reverse: 5'-AATGCTGCAGGAACTGGTG-3'
LATS1	Forward:5'-AGCTTGTGGTGGAATGTGAA-3'
	Reverse: 5'-TGGGACAACCTCCTTTCTTG-3'
YAP	Forward :5'-GCAACTCCAACCAGCAGCAACA-3'
	Reverse: 5'-CGCAGCCTCTCCTTCTCCATCTG-3'
USP9X	Forward:5'-GCATGTCAGCGATTTTTCCG-3'
	Reverse: 5'-CACATAGCTCCACCAGACGATG-3'
AMOT (p130)	Forward:5'-CTTGATGGCCAATAAGCGTTGCCT -3'
	Reverse: 5'-GCAAGCCTGATCCAGCATTGGAAA -3'
AMOT (p80)	Forward:5'-GAGTTCCTGGCTGACAATGG-3'
	Reverse: 5'-CCTTCAGGGAGCTGCTAAGA-3'
AREG	Forward:5'-TCACTTTCCGTCTTGTTTTGG-3'
	Reverse: 5'-CGGGAGCCGACTATGACTAC-3'
Cyr61	Forward:5'-TATTCACAGGGTCTGCCCTC-3'
	Reverse: 5'-AACGAGGACTGCAGCAAAA-3'
CTGF	Forward:5'-TAGGCTTGGAGATTTTGGGA-3'
	Reverse: 5'-GGTTACCAATGACAACGCCT-3'
Survivin	Forward:5'-CTTTCTCCGCAGTTTCCTCA-3'
	Reverse: 5'-TTGGTGAATTTTTGAAACTGGA-3'
ANKRD1	Forward:5'-GTGTAGCACCAGATCCATCG-3'
	Reverse: 5'-CGGTGAGACTGAACCGCTAT-3'
